# Cinnamic Acid Is Partially Involved in Propolis Immunomodulatory Action on Human Monocytes

**DOI:** 10.1155/2013/109864

**Published:** 2013-05-16

**Authors:** Bruno José Conti, Michelle Cristiane Búfalo, Marjorie de Assis Golim, Vassya Bankova, José Maurício Sforcin

**Affiliations:** ^1^Department of Microbiology and Immunology, Biosciences Institute, UNESP, 18618-970 Botucatu, SP, Brazil; ^2^Flow Cytometry Laboratory, Botucatu Blood Center, Medical School, UNESP, 18618-970 Botucatu, SP, Brazil; ^3^Institute of Organic Chemistry with Centre of Phytochemistry, Bulgarian Academy of Sciences, 1113 Sofia, Bulgaria

## Abstract

Propolis is a beehive product used in traditional medicine due to its biological properties. It shows a complex chemical composition including phenolics, such as cinnamic acid (Ci). The mechanisms of action of propolis have been the subject of research recently; however, the involvement of Ci on propolis activity was not investigated on immune cells. Ci effects were evaluated on human monocytes, assessing the expression of Toll-like receptors (TLRs), HLA-DR, and CD80. Cytokine production (TNF-**α** and IL-10) and the fungicidal activity of monocytes were evaluated as well. Data showed that Ci downregulated TLR-2, HLA-DR, and CD80 and upregulated TLR-4 expression by human monocytes. High concentrations of Ci inhibited both TNF-**α** and IL-10 production, whereas the same concentrations induced a higher fungicidal activity against *Candida albicans*. TNF-**α** and IL-10 production was decreased by blocking TLR-4, while the fungicidal activity of monocytes was not affected by blocking TLRs. These results suggest that Ci modulated antigen receptors, cytokine production, and the fungicidal activity of human monocytes depending on concentration, and TLR-4 may be involved in its mechanism of action. Ci seemed to be partially involved in propolis activities.

## 1. Introduction

Propolis is a hive product, collected by honeybees from buds and leaves of trees and plants, mixed with pollen, wax, and bee enzymes. Its chemical composition is complex and more than 300 compounds have been identified, including phenolic acids, terpenes, several esters, and flavonoids [1]. The main phenolics found in Brazilian propolis are cinnamic, caffeic, ferulic, and *p*-coumaric acids [2, 3].

Herein, a possible immunomodulatory action of cinnamic acid (Ci) was investigated, since it has an important role in the synthesis of other compounds, such 3,5-diprenyl-4-hydroxycinnammic acid (artepillin C), which has been reported to exhibit anticancer effects [4]. Propolis, caffeic acid phenethyl ester (CAPE), and artepillin C have been shown to exert immunosuppressive functions on T-lymphocyte subsets but paradoxically activate macrophages [5].

Toll-like receptors (TLR) recognize various conserved pathogen-associated molecular patterns (PAMPs) [6], playing an essential role in host innate immunity with further activation of adaptive immunity [7]. TLR-2 and TLR-4 are transmembrane proteins, showing a domain with a leucine-rich repeat in their extracellular region, which allows the recognition of various PAMPs. TLR-2 recognizes, for example, components from Gram-positive bacteria and zymosan, while TLR-4 recognizes Gram-negative bacteria lipopolysaccharide (LPS). Human antigen-presenting cells (APCs) exhibit HLA-DR molecules, responsible for presenting peptides, and CD80 (B7-1) which acts as a costimulatory molecule for T-cells activation. Moreover, after activation and signal transduction, signaling cascades may activate transcription factors, which in turn lead to the gene expression of proinflammatory cytokines, chemokines, and antimicrobial peptides [8, 9].

Propolis immunomodulatory action has been widely investigated in mice [3, 10]; however, little is known concerning propolis action on human cells. Thus, this work evaluated Ci effects on TLR-2, TLR-4, HLA-DR, and CD80 expression, pro- and anti-inflammatory cytokine production (TNF-*α* and IL-10, resp.), and on the fungicidal activity of human monocytes, in order to understand its effects on the initial events of the immune response in humans and to investigate its involvement in propolis action. The role of TLR-2 and TLR-4 on Ci action was also investigated, as possible Ci ligands.

## 2. Material and Methods

### 2.1. Propolis Composition and Cinnamic Acid

Propolis was produced by Africanized honeybees (*Apis mellifera* L.) in the apiary located on Lageado Farm, UNESP (Brazil). After freezing, propolis was extracted for 24 h with 70% ethanol (1 : 10, w/v) at room temperature. The extract was evaporated to dryness, and approximately 5 mg of the residue was mixed with 75 mL of dry pyridine and 25 mL bis(trimethylsilyl)trifluoracetamide (BSTFA), heated at 80°C for 20 min, and analyzed by gas chromatography-mass spectrometry analysis (GC-MS).

GC-MS analysis was performed with a Hewlett Packard Gas Chromatograph 5890 Series II Plus linked to Hewlett Packard 5972 mass spectrometer system equipped with a 23 m long, 0.25 mm id, and 0.5 mm film thickness HP5-MS capillary column. The temperature was programmed from 100°C to 310°C at a rate of 5°C·min^−1^. Helium was used as a carrier gas, flow rate 0.7 mL/min, split ratio 1 : 80, injector temperature 280°C, and ionization voltage 70 eV. The identification was accomplished using computer searches on a NIST98 MS data library. In some cases, when identical spectra have not been found, only the structural type of the corresponding component was proposed on the basis of its mass-spectral fragmentation. If available, reference compounds were cochromatographed to confirm GC retention times. The components of ethanol extracts of propolis were determined by considering their areas as percentage of the total ion current. Some components remained unidentified because of the lack of authentic samples and library spectra of the corresponding compounds.

Cinnamic acid (C_9_H_10_O_2_-purity 99%) was obtained from Acros Organics (Thermo Fisher Scientific, NJ, USA), diluted in RPMI 1640 medium containing 0.1 g/L of L-glutamine, 2.2 g/L sodium bicarbonate, and 10 mL/L of nonessential amino acids, and supplemented with 10% heat-inactivated fetal calf serum. Dilutions were performed to obtain 5, 10, 25, 50, and 100 *μ*g/mL of cinnamic acid in the cell culture.

### 2.2. Human Monocytes Culture

After approval by the Ethics Committee (CEP 3442-2010), heparinized venous blood was obtained from 10 healthy adult volunteers. Peripheral blood mononuclear cells (PBMC) were isolated by density gradient centrifuging on Histopaque (density = 1.077) (Sigma Chemical Co., St. Louis, MO, USA). Briefly, 5 mL of heparinized blood was mixed with an equal volume of RPMI-1640 tissue culture medium (Gibco Laboratories, Grand Island, NY, USA) containing 2 mM L-glutamine, 10% heat-inactivated fetal calf serum, 20 mM HEPES, and 40 mg/L gentamicin. Samples were layered over 4 mL Histopaque in a 15 mL conical Falcon tube. After centrifuging at 300 g for 30 min at room temperature, the interface layer of PBMC was carefully aspirated and washed twice with RPMI 1640 medium.

Monocytes were counted using 0.02% neutral red for 10 minutes at 37°C. Cell suspension was adjusted to 1 × 10^6^ cells/mL, and 500 *μ*L was added in a 24-welled plate and incubated at 37°C at 5% CO_2_ for 2 h. Afterwards, nonadherent cells were removed and monocytes were incubated with different concentrations of Ci (5, 10, 25, 50, and 100 *μ*g/mL) at 37°C for 18 h to evaluate its possible cytotoxic effect.

### 2.3. Cell Viability

Cell viability was assessed using the 3-(4,5-dimethylthiazol-2-yl-)2,5-diphenyltetrazolium bromide (MTT) colorimetric assay with some modifications [11]. After 18 h incubation with Ci, culture medium was replaced with 300 *μ*L of 1 mg/mL MTT and incubated for 3 h. Subsequently, the cell medium was aspirated, and 200 *μ*L of dimethyl sulfoxide was added to the wells to dissolve the insoluble purple formazan product into a colored solution. The absorbance was measured at 540 nm using a microplate reader.

### 2.4. TLR-2, TLR-4, HLA-DR, and CD80 Determination by Flow Cytometry

After Ci treatment by 18 h, human monocytes were evaluated by TLR-2, TLR-4, HLA-DR, and CD80 expression by flow cytometry analysis. Monocytes (1 × 10^6^ cells/mL) were distributed (500 *μ*L) into polystyrene tubes for cytometric analysis (BD Labware, San Jose, CA, USA). Cells were washed and incubated with fluorescein isothiocyanate (FITC)-, streptavidin-phycoerythrin (PECy7)-, and phycoerythrin (PE)-conjugated monoclonal antibodies (50 *μ*g/mL), as follows: CD14-PECy7, TLR-2-FITC, TLR-4-PE, HLA-DR-FITC, and CD80-PE (Biolegend Inc., San Diego, CA, USA). Isotype control was performed according to the experimental protocol.

After incubation for 20 min at 4°C, cells were analyzed using an FAC SCalibur flow cytometer (Becton Dickinson, San Jose, CA, USA). Data (an average of 10.000 events per sample) were analyzed with the CELL QUEST Software (Cell Quest Software, San Jose, CA, USA).

### 2.5. Cytokine Production

TNF-*α* and IL-10 production was determined using the supernatants of cell cultures and the enzyme-linked immunosorbent assay (ELISA), according to the manufacturer's instructions (BD Biosciences, USA). Briefly, a 96-well flat-bottom Nunc MaxiSorp (Nunc/Apogent, USA) was coated with a capture antibody specific to each cytokine. The plate was washed and blocked before 100 *μ*L of the supernatants, and serially diluted specific standards were added to the respective wells. Following a series of washing, the captured cytokine was detected using the specific conjugated detection antibody. The substrate reagent was added into each well, and, after color development, the plate was read at 450 nm, using an ELISA plate reader [12].

### 2.6. Fungicidal Activity

Monocytes (2 × 10^5^ cells/mL) were dispensed into 96-well flat-bottom plates and activated with Ci at different concentrations (5, 10, 25, 50, and 100 *μ*g/mL) for 18 h at 37°C and 5% CO_2_. Cultures were washed and then challenged for with 100 *μ*L of a *Candida albicans* suspension (ATCC 5314), containing 10 × 10^5^ yeasts/mL (ratio monocyte/fungus = 1 : 5) prepared in RPMI 1640 plus 10% heat-inactivated fetal calf serum. After 1 h at 37°C, cocultures were harvested by aspiration and wells were washed with sterile distilled water to lyse monocytes. Each well washing resulted in a final volume of 2.0 mL, and 0.1 mL was plated in triplicates on supplemented brain-heart infusion (BHI) agar medium (Difco Laboratories, Detroit, MI., USA). Plates were incubated at 35°C in sealed plastic bags to prevent drying. After 24 h, the number of colony forming units (CFU) per plate was counted, and plates containing only monocytes-fungus cocultures were considered as experimental plates, and those plated with the inoculum alone at the beginning were used as control. Fungicidal activity percentage was determined by the following formula:

(1) Fungicidal  Activity  (%)  =1−mean  CFU  recovered  on⁡  experimental  platesmean  CFU  recovered  on⁡  control  plates   ×100.

### 2.7. Role of TLR-2 and TLR-4 on Cytokine Production and Fungicidal Activity of Monocytes

In an attempt to identify possible ligands of Ci, TLR-2 and TLR-4 were blocked before its addition. For cytokine determination, cells were pre-incubated with monoclonal antibodies (anti-TLR-2 and anti-TLR-4; 50 *μ*g/mL—Biolegend, San Diego, CA, USA) for 1 h at 37°C and treated with different concentration of Ci (5, 10, 25, 50, and 100 *μ*g/mL) for 18 h. Supernatants of cell cultures were used for cytokine determination by ELISA.

As to the fungicidal activity, monocytes (2 × 10^5^ cells) were preincubated (1 h at 37°C) with monoclonal antibodies anti-TLR-2 and anti-TLR-4 and treated with different concentration of Ci for 18 h, as described above. Cells were then challenged with a *Candida albicans* suspension containing 10 × 10^5^ yeasts using the same ratio monocyte/fungus described before for 1 h at 37°C. After incubation, the fungicidal activity was determined as previously described.

### 2.8. Statistical Analysis

Data were analyzed using the INSTAT 3.05 software (GraphPad, San Diego, CA, USA). Analysis of variance (ANOVA) was employed, followed by Tukey test, adopting 0.05 as the significant level.

## 3. Results

### 3.1. Propolis Chemical Composition

Our propolis sample was analyzed by GC-MS, revealing that its main groups were phenolic compounds (flavonoids, aromatic acids) and triterpenes (Table 1).

### 3.2. Cell Viability

**Figure 1:**
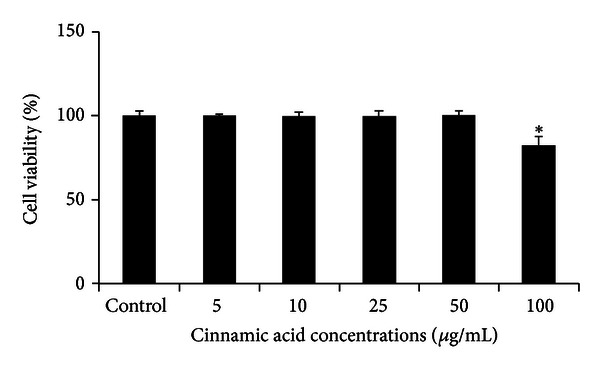
Cell viability (%) of monocytes after incubation with cinnamic acid (5, 10, 25, 50, and 100 *μ*g/mL) or control for 18 h. Data represent mean and standard deviation (*n* = 10). *Significantly different from control (*P* < 0.0001).

Ci did not affect monocytes viability but only the concentration of 100 *μ*g promoted a mild cytotoxic effect (*P* < 0.0001) (Figure 1). Although statistically different from control, we still used 100 *μ*g/mL in the assays, since the cell viability was 82%.

### 3.3. TLR-2, TLR-4, HLA-DR, and CD80 Expression

**Figure 2:**
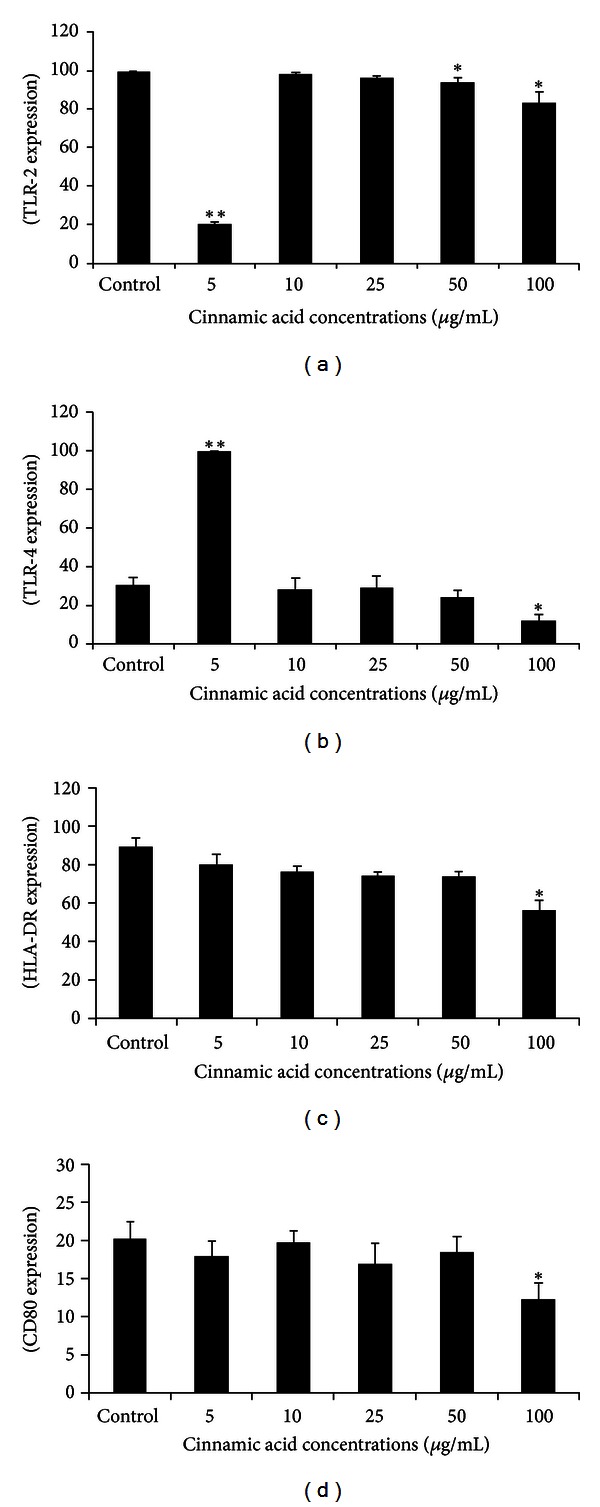
Mean fluorescence intensity (MIF) of TLR-2, TLR-4, HLA-DR, and CD80 expression by human monocytes incubated with cinnamic acid (5, 10, 25, 50, and 100 *μ*g/mL) for 18 h. Data represent mean and standard deviation (*n* = 10). *Significantly different from control (*P* < 0.01), **significantly different from control (*P* < 0.001).

The results of flow cytometry are shown as mean fluorescence intensity (MFI), which is the amount of surface-binding sites to which the antibodies are bound. Ci downregulated TLR-2 expression by human monocytes using 5 (*P* < 0.001), 50, and 100 *μ*g/mL (*P* < 0.01). On the other hand, TLR-4 expression was increased after monocytes incubation with Ci (5 *μ*g/mL) (*P* < 0.001), while an inhibitory effect was seen using 100 *μ*g/mL (*P* < 0.01). HLA-DR was inhibited after monocytes treatment with Ci in all concentrations. CD80 was also inhibited by Ci (100 *μ*g/mL—*P* < 0.01) (Figure 2).

### 3.4. Cytokine Production and the Role of TLR-2 and TLR-4

**Figure 3:**
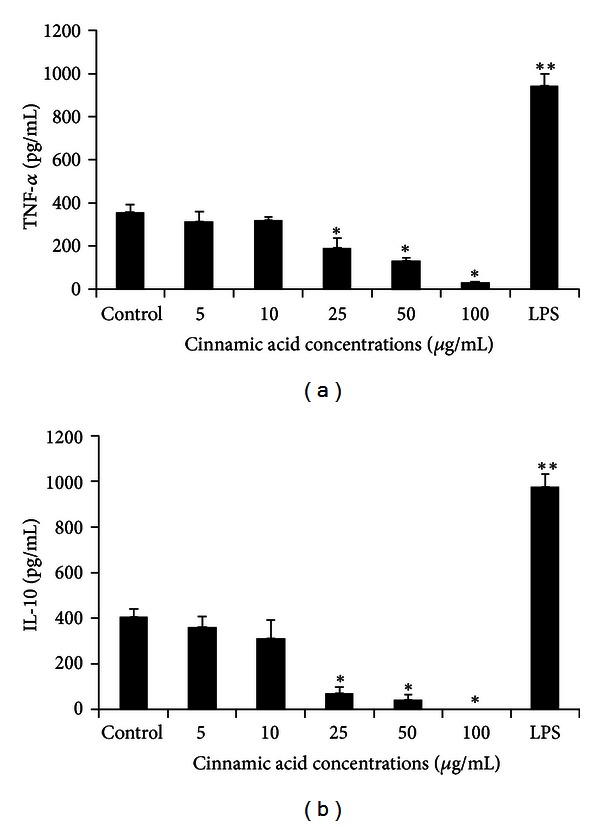
TNF-*α* and IL-10 production (pg/mL) by monocytes incubated with cinnamic acid (5, 10, 25, 50, and 100 *μ*g/mL), control, or LPS (10 *μ*g/mL) for 18 h. Data represent mean and standard deviation (*n* = 10). *Significantly different from control (*P* < 0.01), **significantly different from control (*P* < 0.001).

When cytokine production was evaluated without blocking antibodies, both TNF-*α* and IL-10 production was inhibited by Ci using 25, 50, and 100 *μ*g/mL (*P* < 0.01) (Figure 3).

**Figure 4:**
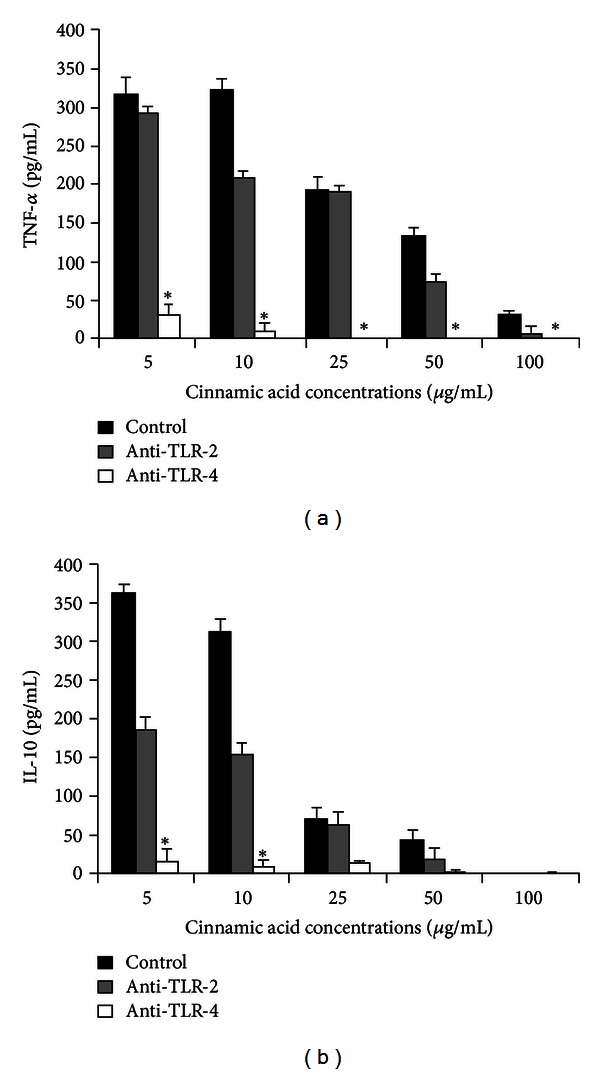
TNF-*α* and IL-10 production (pg/mL) by human monocytes treated or not with anti-TLR-2 or anti-TLR-4 and incubated with cinnamic acid (5, 10, 25, 50, and 100 *μ*g/mL) for 18 h. Data represent mean and standard deviation (*n* = 10). *Significantly different from control of the respective concentration (*P* < 0.001).

Using anti-TLR-4 before incubation with cinnamic acid, a reduced TNF-*α* production was seen at all concentrations of Ci. A significant reduction in IL-10 production was seen after incubating monocytes with anti-TLR-4 and further with 5 and 10 *μ*g/mL of cinnamic acid (*P* < 0.001) (Figure 4). These data suggest the involvement of TLR-4 at least in part in cinnamic acid action in human monocytes.

### 3.5. Fungicidal Activity and the Role of TLR-2 and TLR-4

**Figure 5:**
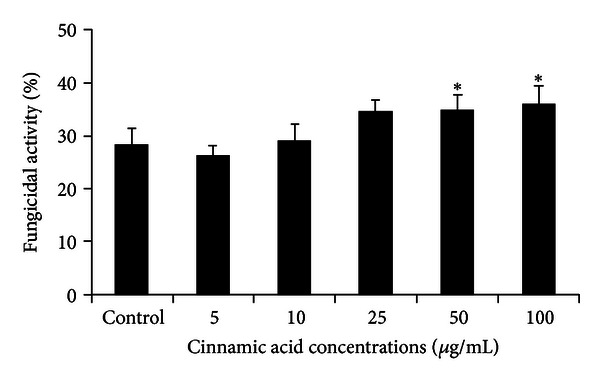
Fungicidal activity (%) of control monocytes or incubated with cinnamic acid (5, 10, 25, 50, and 100 *μ*g/mL) and challenged with *C. albicans* (ratio monocytes/fungus = 1 : 5) for 2 h. Data represent mean and standard deviation (*n* = 10). *Significantly different from control (*P* < 0.01).

An increased fungicidal activity of human monocytes against *C. albicans* was seen after incubation with Ci (50 and 100 *μ*g/mL—*P* < 0.01). Although the concentration 25 *μ*g/mL was not statistically different from control, an increased fungicidal activity was also observed (Figure 5).

**Figure 6:**
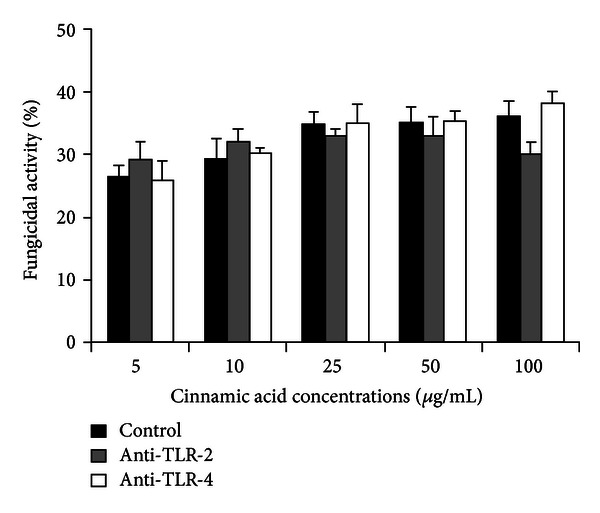
Fungicidal activity (%) of human monocytes treated or not with anti-TLR-2 or anti-TLR-4, incubated with cinnamic acid (5, 10, 25, 50, and 100 *μ*g/mL), and challenged with *C. albicans* (ratio monocytes/fungus = 1 : 5) for 2 h. Data represent mean and standard deviation (*n* = 10).

The fungicidal activity of human monocytes was not affected using anti-TLR-2 and anti-TLR-4 before incubation with Ci, and the means were similar in each concentration to the respective control (Figure 6).

## 4. Discussion

In mice, experimental works of our group revealed that propolis increased proinflammatory cytokine production and TLR-2 and TLR-4 expression by peritoneal macrophages and spleen cells, suggesting that propolis activated the initial steps of the immune response, modulating the mechanisms of the innate immunity [10, 13].

In the last years, the investigation of isolated compounds responsible for propolis action has increased, and CAPE has been the most extensively studied compound in propolis [14]. Cinnamic acid derivatives have been investigated as well, demonstrating anticancer activities, tissue factor induced by TNF-*α* in endothelial cells, and luminol-enhanced chemiluminescence of neutrophils [15–17].

However, most of the articles have been published using experimental animals, mainly mice or rats, and little is known concerning propolis effect on human cells. Herein, we wish to present the effects of cinnamic acid on human monocytes. 

Constitutively, monocytes express about 80% TLR-2 and 40–80% TLR-4; furthermore, monocytes exhibit an increased expression of HLA-DR in relation to CD80/86 [18]. These markers can be detected in immature dendritic cells and monocytes and may be modulated in response to different stimuli [19]. One may verify that Ci inhibited the expression of TLR-2, HLA-DR, and CD80 and stimulated the expression of TLR-4 by human monocytes. According to Sforcin and Bankova [20], it has been reported that propolis shows a potential for the development of new drugs. In an attempt to verify whether Ci could be responsible for propolis action, we also verified propolis effects in the same experimental protocols, observing that propolis upregulated TLR-4 and CD80 expression by human monocytes (unpublished data). Ci also upregulated TLR-4 expression, although it downregulated TLR-2, HLA-DR, and CD80 expression at noncytotoxic concentrations, suggesting that it may be at least in part involved in propolis action.

TNF-*α* and IL-10 production by such cells was inhibited by high concentrations of Ci, while an increased fungicidal activity was seen against *C. albicans*. Although TNF-*α* is important to activate monocytes and macrophages while IL-10 may suppress these cells, one may speculate that the fungicidal activity of monocytes incubated with Ci may have involved other effector mechanisms, such as nitrogen and oxygen reactive intermediates after interaction with the fungus. It has been reported that hydrogen peroxide (H_2_O_2_) could be efficient in the first hours after phagocytosis, while nitric oxide (NO) could be important either in the beginning or late in the antimicrobial activity of macrophages [21]. Although these mediators were not determined herein, the highest concentrations of Ci could have induced both H_2_O_2_ and NO, increasing the fungicidal activity of monocytes.

It is known that phenolic compounds can interact with lipids in cell membrane unspecifically, based essentially on physical adsorption [22]. However, it is not known whether propolis compounds enter unspecifically or interact with cell receptors, exerting its biological activities. Data showed that Ci upregulated TLR-4 expression by human monocytes; thus, in order to investigate the mechanism of action of Ci, assays were carried out using monoclonal antibodies anti-TLR-2 or anti-TLR-4. The production of TNF-*α* and IL-10 was inhibited only by blocking TLR-4, whereas the fungicidal activity of monocytes was not affected, suggesting that Ci may interact with TLR-4 and trigger the activation of transcription nuclear factors, which in turn induce cytokines gene expression. Our data are in agreement with Nakaira-Takahagi [23], who incubated human monocytes with anti-TLR-2 or anti-TLR-4 and challenged the cells with gp43 isolated from *Paracoccidioides brasiliensis*. TNF-*α* was not affected by blocking TLR-2, but low levels were seen using anti-TLR-4, suggesting the role of TLR-4 in TNF-*α* production by this fungus. On the contrary, [24] verified that blocking TLR-2 on human mononuclear cells inhibited TNF-*α* production induced by *C. albicans*, suggesting that the induction of proinflammatory cytokines by *Candida* species is partially, but not exclusively, mediated by TLR-2. 

Recently, we verified that ethanolic extract of propolis induced TNF-*α* and IL-10 production by human monocytes, using the same experimental protocols (unpublished data). Ci inhibited both cytokine production; nevertheless, propolis and Ci inhibited cytokine production by blocking TLR-4. Propolis and Ci enhanced the fungicidal activity of monocytes using the same concentrations, suggesting that Ci may be partially involved in propolis action. 

Natural products may modulate the action of APCs, favoring or inhibiting initial steps of an immune response. Taken together, our findings indicate that Ci displayed an immunomodulatory action depending on concentration and seemed to be partially involved in propolis action; moreover, TLR-4 may be involved in its action. Further investigation should confirm Ci role on propolis action in other experimental approaches, as well as the synergism between propolis constituents.

## Figures and Tables

**Table 1:** Relative percentages of compounds, determined by GC-MS, from ethanolic extract of Brazilian propolis.

Component	Retention time (min)	% of total
Benzoic acid	9.3	0.193
Dihydrocinnamic acid	14	2.180
Dihydrocinnamic acid	22	0.860
Coumaric acid	26.5	0.382
Cafeic acid	28.6	0.297
Prenyl-*p*-coumaric acid	32.5	6.560
Flavonoids	35.8	1.142
Artepillin C	37.7	16.750
Trihydroxymethoxy flavonon	40.5	0.666
Tetrahydroxy flavonon	40.8	0.228
Triterpene	47.6	0.777
Triterpene	51	0.309
